# Poly[aqua­{μ_3_-5-[(pyridin-2-ylmeth­yl)amino]­isophthalato-κ^5^ *N*,*N*′:*O* ^1^,*O* ^1′^:*O* ^3^}cobalt(II)]

**DOI:** 10.1107/S1600536811050318

**Published:** 2011-11-30

**Authors:** Xiao-Hong Zhu, Xiao-Chun Cheng

**Affiliations:** a Faculty of Life Science and Chemical Engineering, Huaiyin Institute of Technology, Huaian 223003, People’s Republic of China

## Abstract

In the title polymer, {[Co(C_14_H_10_N_2_O_4_)(H_2_O)]·3.5H_2_O}_*n*_, the Co^2+^ ion is coordinated by three carboxyl­ate O atoms from two 5-[(pyridin-2-ylmeth­yl)amino]­isophthalate anions, two N atoms from a (pyridin-2-ylmeth­yl)amino group and an O atom from a water mol­ecule, furnishing a distorted CoO_4_N_2_ octa­hedral geometry. Each anion acts as a μ_3_-bridge, linking cobalt ions into a two-dimensional layer parallel to (100). The asymmetric unit also contains three and a half solvent water mol­ecules, which could not be modeled. Therefore, the diffraction contribution of the solvent water mol­ecules was removed by the subroutine SQUEEZE in *PLATON* [Spek (2009). *Acta Cryst.* D**65**, 148–155]. The crystal structure is stabilized by O—H⋯O hydrogen bonds in which the coordinated water mol­ecule acts as donor and the carboxyl­ate O atoms as acceptors.

## Related literature

For related structures, see: Kuai *et al.* (2011 ▶).
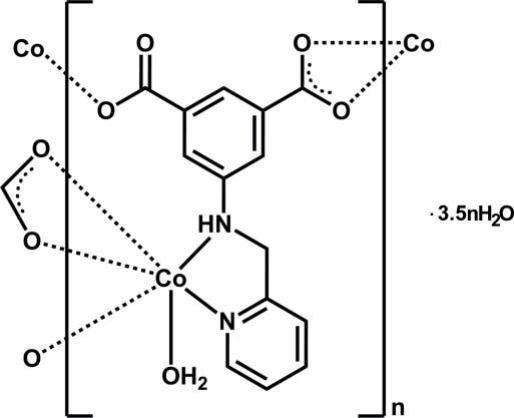


## Experimental

### 

#### Crystal data

[Co(C_14_H_10_N_2_O_4_)(H_2_O)]·3.5H_2_O
*M* *_r_* = 410.24
Monoclinic, 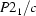

*a* = 11.0926 (11) Å
*b* = 9.8735 (10) Å
*c* = 17.4317 (15) Å
β = 116.533 (5)°
*V* = 1708.1 (3) Å^3^
*Z* = 4
Mo *K*α radiation
μ = 1.05 mm^−1^
*T* = 293 K
0.20 × 0.20 × 0.20 mm

#### Data collection

Bruker SMART APEXII CCD diffractometer
Absorption correction: multi-scan (*SADABS*; Sheldrick, 1996 ▶) *T*_min_ = 0.821, *T*_max_ = 0.821
11993 measured reflections
4259 independent reflections
3809 reflections with *I* > 2σ(*I*)
*R*_int_ = 0.021

#### Refinement

*R*[*F* ^2^ > 2σ(*F* ^2^)] = 0.045
*wR*(*F* ^2^) = 0.124
*S* = 1.11
4259 reflections
199 parameters
H-atom parameters constrained
Δρ_max_ = 0.81 e Å^−3^
Δρ_min_ = −0.43 e Å^−3^

### 

Data collection: *APEX2* (Bruker, 2008 ▶); cell refinement: *SAINT* (Bruker, 2008 ▶); data reduction: *SAINT*; program(s) used to solve structure: *SHELXS97* (Sheldrick, 2008 ▶); program(s) used to refine structure: *SHELXL97* (Sheldrick, 2008 ▶); molecular graphics: *DIAMOND* (Brandenburg, 2000 ▶); software used to prepare material for publication: *SHELXTL* (Sheldrick, 2008 ▶) and *PLATON* (Spek, 2009 ▶).

## Supplementary Material

Crystal structure: contains datablock(s) I, global. DOI: 10.1107/S1600536811050318/pv2477sup1.cif

Structure factors: contains datablock(s) I. DOI: 10.1107/S1600536811050318/pv2477Isup2.hkl

Supplementary material file. DOI: 10.1107/S1600536811050318/pv2477Isup3.cdx

Additional supplementary materials:  crystallographic information; 3D view; checkCIF report

## Figures and Tables

**Table 1:** Selected bond lengths (Å)

Co1—O3^i^	1.9999 (19)
Co1—N2^ii^	2.090 (2)
Co1—O5	2.110 (2)
Co1—O1	2.132 (2)
Co1—O2	2.195 (2)
Co1—N1^ii^	2.275 (2)

Symmetry codes: (i) 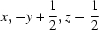; (ii) 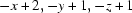.

**Table 2:** Hydrogen-bond geometry (Å, °)

*D*—H⋯*A*	*D*—H	H⋯*A*	*D*⋯*A*	*D*—H⋯*A*
O5—H5*WA*⋯O4^i^	0.87	2.25	2.946 (3)	137
O5—H5*WA*⋯O1^iii^	0.87	2.42	3.047 (3)	130
O5—H5*W*⋯O4^iv^	0.93	1.90	2.819 (3)	168

Symmetry codes: (i) 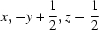; (iii) 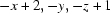; (iv) 
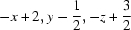
.

**Fig. 1.**
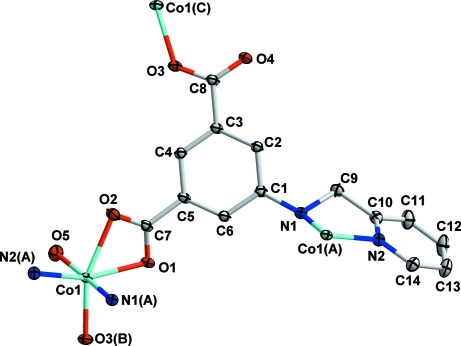
: The coordination environment of Co ion in the title complex with the ellipsoids drawn at the 30% probability level. The hydrogen atoms have been omitted for clarity. Symmetry codes: A = -x + 2, -y + 1, -z + 1; B = x, -y + 1/2, z - 1/2; C = x, -y + 1/2, z + 1/2.

## supplementary crystallographic information

### Comment

5-(Pyridin-2-ylmethyl)aminoisophthalic acid possesses some peculiar features in the assembly of coordination compounds due to its unique molecular structure. Herein, we report the crystal structure of the title complex. The asymmetric unit consists of one cobalt ion, one 5-(pyridin-2-ylmethyl)aminoisophthalate anion, one coordinated water molecule, and 3.5 lattice water molecules. However, the solvent water molecules could not be modeled as discrete atomic sites. We employed SQUEEZE subroutine in PLATON (Spek, 2009) to exclude the diffraction contribution of the solvent water molecules.

In the title polymer, each Co ion is coordinated by three carboxylate O atoms from two different 5-(pyridin-2-ylmethyl)aminoisophthalate anions,  two N atoms from (pyridin-2-ylmethyl)amino group and one O atom from a coordinated water molecule, to furnish a distorted CoO_4_N_2_ octahedral geometry (Fig. 1). Each anion acts as a µ_3_-bridge, linking cobalt ions to form a two-dimensional layer. In the crystal structure, there exist O—H···O hydrogen bonds (Table 1). Coordinated water molecule and carboxylate oxygen atoms act as donors or acceptors in the formation of these hydrogen bonding interactions.

### Experimental

A mixture of cobalt nitrate hexahydrate (58.2 mg, 0.2 mmol), 5-(pyridin-2-ylmethyl)aminoisophthalic acid (54.4 mg, 0.2 mmol), and 3 ml *N*,*N*-dimethylformamide in H_2_O (12 ml) was sealed in a 16 ml Teflon-lined stainless steel container and heated to 373 K for 3 days. After cooling the container to the room temperature, red block crystals of the title complex were obtained.

### Refinement

The hydrogen atoms bonded to C atoms were included in geometrically idealized positions and constrained to ride on their parent atoms, with C—H = 0.93 and 0.97 Å for aryl and methylene H-atoms with *U*_iso_(H) = 1.2*U*_eq_(C). The hydrogen atoms bonded to N1 and O5 were located from a difference Fourier map and fixed at those positions  with *U*_iso_(H) = 1.2*U*_eq_(N or O)]. The structure contains three and a half molecules of water of hydration, which could not be modeled as discrete atomic sites. We employed SQUEEZE in PLATON (Spek, 2009) to remove the diffraction contribution of the solvent water molecules.

### Crystal data

**Table:**

[Co(C_14_H_10_N_2_O_4_)(H_2_O)]·3.5H_2_O	*F*(000) = 846
*M**_r_* = 410.24	*D*_x_ = 1.593 Mg m^−^^3^
Monoclinic, *P*2_1_/*c*	Mo *K*α radiation, λ = 0.71073 Å
Hall symbol: -P 2ybc	Cell parameters from 6314 reflections
*a* = 11.0926 (11) Å	θ = 2.4–28.4°
*b* = 9.8735 (10) Å	µ = 1.05 mm^−^^1^
*c* = 17.4317 (15) Å	*T* = 293 K
β = 116.533 (5)°	Block, red
*V* = 1708.1 (3) Å^3^	0.20 × 0.20 × 0.20 mm
*Z* = 4	

### Data collection

**Table:**

Bruker SMART APEXII CCD diffractometer	4259 independent reflections
Radiation source: fine-focus sealed tube	3809 reflections with *I* > 2σ(*I*)
graphite	*R*_int_ = 0.021
φ and ω scans	θ_max_ = 28.4°, θ_min_ = 2.1°
Absorption correction: multi-scan (*SADABS*; Sheldrick, 1996)	*h* = −14→12
*T*_min_ = 0.821, *T*_max_ = 0.821	*k* = −13→10
11993 measured reflections	*l* = −22→23

### Refinement

**Table:**

Refinement on *F*^2^	Primary atom site location: structure-invariant direct methods
Least-squares matrix: full	Secondary atom site location: difference Fourier map
*R*[*F*^2^ > 2σ(*F*^2^)] = 0.045	Hydrogen site location: inferred from neighbouring sites
*wR*(*F*^2^) = 0.124	H-atom parameters constrained
*S* = 1.11	*w* = 1/[σ^2^(*F*_o_^2^) + (0.0407*P*)^2^ + 3.8921*P*] where *P* = (*F*_o_^2^ + 2*F*_c_^2^)/3
4259 reflections	(Δ/σ)_max_ = 0.001
199 parameters	Δρ_max_ = 0.81 e Å^−^^3^
0 restraints	Δρ_min_ = −0.43 e Å^−^^3^

### Special details

**Table:**

Geometry. All e.s.d.'s (except the e.s.d. in the dihedral angle between two l.s. planes) are estimated using the full covariance matrix. The cell e.s.d.'s are taken into account individually in the estimation of e.s.d.'s in distances, angles and torsion angles; correlations between e.s.d.'s in cell parameters are only used when they are defined by crystal symmetry. An approximate (isotropic) treatment of cell e.s.d.'s is used for estimating e.s.d.'s involving l.s. planes.
Refinement. Refinement of *F*^2^ against ALL reflections. The weighted *R*-factor *wR* and goodness of fit *S* are based on *F*^2^, conventional *R*-factors *R* are based on *F*, with *F* set to zero for negative *F*^2^. The threshold expression of *F*^2^ > σ(*F*^2^) is used only for calculating *R*-factors(gt) *etc*. and is not relevant to the choice of reflections for refinement. *R*-factors based on *F*^2^ are statistically about twice as large as those based on *F*, and *R*- factors based on ALL data will be even larger.

### Fractional atomic coordinates and isotropic or equivalent isotropic displacement parameters (Å^2^)

**Table:**

	*x*	*y*	*z*	*U*_iso_*/*U*_eq_	
C1	0.8386 (3)	0.5358 (3)	0.59337 (16)	0.0210 (5)	
C2	0.8790 (3)	0.5330 (3)	0.68135 (17)	0.0225 (5)	
H2	0.8344	0.5858	0.7048	0.027*	
C3	0.9868 (3)	0.4503 (3)	0.73445 (16)	0.0217 (5)	
C4	1.0543 (3)	0.3722 (3)	0.70059 (16)	0.0214 (5)	
H4	1.1273	0.3195	0.7364	0.026*	
C5	1.0122 (3)	0.3726 (3)	0.61166 (16)	0.0216 (5)	
C6	0.9043 (3)	0.4529 (3)	0.55925 (17)	0.0231 (5)	
H6	0.8752	0.4514	0.5003	0.028*	
C7	1.0840 (3)	0.2913 (3)	0.57301 (17)	0.0232 (5)	
C8	1.0286 (3)	0.4465 (3)	0.82983 (17)	0.0242 (5)	
C9	0.6291 (3)	0.6616 (3)	0.55952 (19)	0.0291 (6)	
H9A	0.6685	0.7056	0.6151	0.035*	
H9B	0.5806	0.5819	0.5630	0.035*	
C10	0.5346 (3)	0.7568 (3)	0.49261 (18)	0.0261 (5)	
C11	0.3962 (3)	0.7546 (3)	0.4660 (2)	0.0375 (7)	
H11	0.3587	0.6926	0.4894	0.045*	
C12	0.3154 (3)	0.8459 (4)	0.4045 (3)	0.0453 (9)	
H12	0.2226	0.8456	0.3856	0.054*	
C13	0.3740 (3)	0.9382 (4)	0.3709 (2)	0.0419 (8)	
H13	0.3216	1.0010	0.3297	0.050*	
C14	0.5124 (3)	0.9341 (3)	0.4002 (2)	0.0333 (6)	
H14	0.5523	0.9954	0.3781	0.040*	
Co1	1.20146 (4)	0.17514 (4)	0.49770 (2)	0.02074 (11)	
N1	0.7352 (2)	0.6226 (2)	0.53540 (15)	0.0239 (5)	
H1N	0.6876	0.5862	0.4822	0.029*	
N2	0.5908 (2)	0.8448 (2)	0.45967 (15)	0.0243 (5)	
O1	1.0210 (2)	0.2537 (2)	0.49556 (13)	0.0298 (4)	
O2	1.2068 (2)	0.2644 (2)	0.61450 (14)	0.0318 (5)	
O3	1.1274 (2)	0.3701 (2)	0.87308 (12)	0.0270 (4)	
O4	0.9694 (3)	0.5200 (3)	0.86000 (15)	0.0454 (6)	
O5	1.1716 (2)	−0.0155 (2)	0.54116 (15)	0.0337 (5)	
H5WA	1.1069	−0.0587	0.4995	0.040*	
H5W	1.1367	−0.0012	0.5799	0.040*	

### Atomic displacement parameters (Å^2^)

**Table:**

	*U*^11^	*U*^22^	*U*^33^	*U*^12^	*U*^13^	*U*^23^
C1	0.0250 (12)	0.0200 (12)	0.0202 (12)	0.0001 (10)	0.0119 (10)	0.0011 (9)
C2	0.0271 (13)	0.0227 (12)	0.0222 (12)	0.0027 (10)	0.0151 (10)	0.0014 (10)
C3	0.0277 (13)	0.0225 (12)	0.0175 (11)	−0.0013 (10)	0.0123 (10)	−0.0010 (9)
C4	0.0258 (12)	0.0208 (12)	0.0190 (11)	0.0020 (10)	0.0113 (10)	0.0015 (9)
C5	0.0289 (13)	0.0210 (12)	0.0196 (12)	−0.0015 (10)	0.0150 (10)	−0.0012 (9)
C6	0.0297 (13)	0.0236 (12)	0.0182 (11)	−0.0003 (10)	0.0127 (10)	−0.0004 (10)
C7	0.0330 (14)	0.0202 (12)	0.0232 (12)	0.0004 (10)	0.0187 (11)	0.0002 (10)
C8	0.0338 (14)	0.0241 (13)	0.0202 (12)	−0.0003 (11)	0.0170 (11)	0.0002 (10)
C9	0.0282 (14)	0.0330 (15)	0.0322 (15)	0.0032 (12)	0.0188 (12)	0.0063 (12)
C10	0.0263 (13)	0.0258 (13)	0.0275 (13)	0.0020 (11)	0.0133 (11)	−0.0011 (11)
C11	0.0260 (14)	0.0339 (16)	0.051 (2)	−0.0013 (12)	0.0157 (14)	0.0028 (14)
C12	0.0244 (15)	0.0425 (19)	0.064 (2)	0.0062 (14)	0.0155 (15)	0.0073 (17)
C13	0.0323 (16)	0.0419 (18)	0.0445 (19)	0.0135 (14)	0.0110 (14)	0.0100 (15)
C14	0.0347 (15)	0.0333 (15)	0.0318 (15)	0.0097 (13)	0.0149 (13)	0.0072 (12)
Co1	0.02195 (19)	0.0267 (2)	0.01563 (17)	0.00285 (14)	0.01024 (13)	0.00087 (13)
N1	0.0257 (11)	0.0274 (11)	0.0203 (10)	0.0046 (9)	0.0117 (9)	0.0020 (9)
N2	0.0241 (11)	0.0259 (11)	0.0234 (11)	0.0032 (9)	0.0110 (9)	0.0016 (9)
O1	0.0366 (11)	0.0365 (11)	0.0191 (9)	0.0087 (9)	0.0150 (8)	−0.0012 (8)
O2	0.0298 (10)	0.0399 (12)	0.0307 (11)	−0.0009 (9)	0.0179 (9)	−0.0085 (9)
O3	0.0306 (10)	0.0348 (11)	0.0181 (9)	0.0023 (9)	0.0131 (8)	0.0022 (8)
O4	0.0668 (16)	0.0505 (15)	0.0279 (11)	0.0300 (13)	0.0293 (12)	0.0095 (10)
O5	0.0338 (11)	0.0296 (11)	0.0398 (12)	−0.0010 (9)	0.0183 (10)	0.0026 (9)

### Geometric parameters (Å, °)

**Table:**

C1—C2	1.393 (4)	C10—C11	1.391 (4)
C1—C6	1.394 (4)	C11—C12	1.381 (5)
C1—N1	1.427 (3)	C11—H11	0.9300
C2—C3	1.402 (4)	C12—C13	1.392 (5)
C2—H2	0.9300	C12—H12	0.9300
C3—C4	1.377 (4)	C13—C14	1.384 (5)
C3—C8	1.514 (3)	C13—H13	0.9300
C4—C5	1.405 (3)	C14—N2	1.343 (4)
C4—H4	0.9300	C14—H14	0.9300
C5—C6	1.385 (4)	Co1—O3^i^	1.9999 (19)
C5—C7	1.488 (3)	Co1—N2^ii^	2.090 (2)
C6—H6	0.9300	Co1—O5	2.110 (2)
C7—O2	1.252 (4)	Co1—O1	2.132 (2)
C7—O1	1.267 (3)	Co1—O2	2.195 (2)
C8—O4	1.242 (3)	Co1—N1^ii^	2.275 (2)
C8—O3	1.265 (3)	N1—Co1^ii^	2.275 (2)
C9—N1	1.468 (3)	N1—H1N	0.9118
C9—C10	1.500 (4)	N2—Co1^ii^	2.090 (2)
C9—H9A	0.9700	O3—Co1^iii^	1.9999 (19)
C9—H9B	0.9700	O5—H5WA	0.8710
C10—N2	1.339 (4)	O5—H5W	0.9279
			
C2—C1—C6	119.1 (2)	C13—C12—H12	120.3
C2—C1—N1	123.6 (2)	C14—C13—C12	118.3 (3)
C6—C1—N1	117.3 (2)	C14—C13—H13	120.8
C1—C2—C3	119.9 (2)	C12—C13—H13	120.8
C1—C2—H2	120.1	N2—C14—C13	122.3 (3)
C3—C2—H2	120.1	N2—C14—H14	118.9
C4—C3—C2	120.7 (2)	C13—C14—H14	118.9
C4—C3—C8	119.9 (2)	O3^i^—Co1—N2^ii^	102.58 (9)
C2—C3—C8	119.4 (2)	O3^i^—Co1—O5	97.84 (9)
C3—C4—C5	119.6 (2)	N2^ii^—Co1—O5	96.50 (9)
C3—C4—H4	120.2	O3^i^—Co1—O1	97.79 (8)
C5—C4—H4	120.2	N2^ii^—Co1—O1	156.61 (9)
C6—C5—C4	119.5 (2)	O5—Co1—O1	91.96 (8)
C6—C5—C7	119.3 (2)	O3^i^—Co1—O2	157.69 (8)
C4—C5—C7	121.1 (2)	N2^ii^—Co1—O2	98.01 (9)
C5—C6—C1	121.1 (2)	O5—Co1—O2	88.21 (9)
C5—C6—H6	119.4	O1—Co1—O2	60.40 (8)
C1—C6—H6	119.4	O3^i^—Co1—N1^ii^	86.81 (8)
O2—C7—O1	119.6 (2)	N2^ii^—Co1—N1^ii^	75.91 (9)
O2—C7—C5	121.1 (2)	O5—Co1—N1^ii^	171.88 (9)
O1—C7—C5	119.2 (2)	O1—Co1—N1^ii^	94.02 (8)
O4—C8—O3	125.3 (3)	O2—Co1—N1^ii^	89.96 (8)
O4—C8—C3	119.4 (3)	C1—N1—C9	116.7 (2)
O3—C8—C3	115.3 (2)	C1—N1—Co1^ii^	117.72 (17)
N1—C9—C10	108.2 (2)	C9—N1—Co1^ii^	102.74 (17)
N1—C9—H9A	110.1	C1—N1—H1N	113.6
C10—C9—H9A	110.1	C9—N1—H1N	102.8
N1—C9—H9B	110.1	Co1^ii^—N1—H1N	101.1
C10—C9—H9B	110.1	C10—N2—C14	119.4 (3)
H9A—C9—H9B	108.4	C10—N2—Co1^ii^	115.93 (18)
N2—C10—C11	121.6 (3)	C14—N2—Co1^ii^	124.6 (2)
N2—C10—C9	116.2 (2)	C7—O1—Co1	91.19 (17)
C11—C10—C9	122.2 (3)	C7—O2—Co1	88.71 (16)
C12—C11—C10	119.0 (3)	C8—O3—Co1^iii^	127.34 (17)
C12—C11—H11	120.5	Co1—O5—H5WA	110.0
C10—C11—H11	120.5	Co1—O5—H5W	108.0
C11—C12—C13	119.4 (3)	H5WA—O5—H5W	103.0
C11—C12—H12	120.3		
			
C6—C1—C2—C3	1.8 (4)	C6—C1—N1—C9	153.4 (3)
N1—C1—C2—C3	−176.3 (2)	C2—C1—N1—Co1^ii^	94.4 (3)
C1—C2—C3—C4	0.4 (4)	C6—C1—N1—Co1^ii^	−83.8 (3)
C1—C2—C3—C8	−179.2 (2)	C10—C9—N1—C1	177.6 (2)
C2—C3—C4—C5	−1.7 (4)	C10—C9—N1—Co1^ii^	47.3 (3)
C8—C3—C4—C5	177.9 (2)	C11—C10—N2—C14	0.7 (4)
C3—C4—C5—C6	0.7 (4)	C9—C10—N2—C14	−179.2 (3)
C3—C4—C5—C7	179.0 (2)	C11—C10—N2—Co1^ii^	−176.5 (2)
C4—C5—C6—C1	1.6 (4)	C9—C10—N2—Co1^ii^	3.6 (3)
C7—C5—C6—C1	−176.7 (2)	C13—C14—N2—C10	−0.5 (5)
C2—C1—C6—C5	−2.8 (4)	C13—C14—N2—Co1^ii^	176.4 (3)
N1—C1—C6—C5	175.4 (2)	O2—C7—O1—Co1	−3.4 (3)
C6—C5—C7—O2	149.9 (3)	C5—C7—O1—Co1	173.6 (2)
C4—C5—C7—O2	−28.4 (4)	O3^i^—Co1—O1—C7	−173.06 (16)
C6—C5—C7—O1	−27.1 (4)	N2^ii^—Co1—O1—C7	−22.6 (3)
C4—C5—C7—O1	154.7 (3)	O5—Co1—O1—C7	88.75 (17)
C4—C3—C8—O4	178.1 (3)	O2—Co1—O1—C7	1.93 (15)
C2—C3—C8—O4	−2.3 (4)	N1^ii^—Co1—O1—C7	−85.74 (17)
C4—C3—C8—O3	0.9 (4)	O1—C7—O2—Co1	3.3 (3)
C2—C3—C8—O3	−179.5 (2)	C5—C7—O2—Co1	−173.6 (2)
N1—C9—C10—N2	−37.2 (4)	O3^i^—Co1—O2—C7	11.2 (3)
N1—C9—C10—C11	142.9 (3)	N2^ii^—Co1—O2—C7	168.46 (17)
N2—C10—C11—C12	−0.3 (5)	O5—Co1—O2—C7	−95.23 (17)
C9—C10—C11—C12	179.6 (3)	O1—Co1—O2—C7	−1.95 (16)
C10—C11—C12—C13	−0.4 (6)	N1^ii^—Co1—O2—C7	92.69 (17)
C11—C12—C13—C14	0.5 (6)	O4—C8—O3—Co1^iii^	9.0 (4)
C12—C13—C14—N2	−0.1 (5)	C3—C8—O3—Co1^iii^	−174.00 (17)
C2—C1—N1—C9	−28.5 (4)		

Symmetry codes: (i) *x*, −*y*+1/2, *z*−1/2; (ii) −*x*+2, −*y*+1, −*z*+1; (iii) *x*, −*y*+1/2, *z*+1/2.

### Hydrogen-bond geometry (Å, °)

**Table:**

*D*—H···*A*	*D*—H	H···*A*	*D*···*A*	*D*—H···*A*
O5—H5WA···O4^i^	0.87	2.25	2.946 (3)	137.
O5—H5WA···O1^iv^	0.87	2.42	3.047 (3)	130.
O5—H5W···O4^v^	0.93	1.90	2.819 (3)	168.

Symmetry codes: (i) *x*, −*y*+1/2, *z*−1/2; (iv) −*x*+2, −*y*, −*z*+1; (v) −*x*+2, *y*−1/2, −*z*+3/2.
